# Effects of the Particle Size of BaTiO_3_ Fillers on Fabrication and Dielectric Properties of BaTiO_3_/Polymer/Al Films for Capacitor Energy-Storage Application

**DOI:** 10.3390/ma12030439

**Published:** 2019-01-31

**Authors:** Lulu Gu, Tao Li, Yongjun Xu, Chenghua Sun, Zhenyu Yang, Deliang Zhu, Deliang Chen

**Affiliations:** 1 College of Materials Science and Engineering, Shenzhen University, Shenzhen 518060, China; lulugu1130@163.com; 2 School of Materials Science and Engineering & School of Chemical Engineering and Energy Technology, Dongguan University of Technology, Dongguan 523808, China; ltnano@hotmail.com (T.L.); hnllxyj@dgut.edu.cn (Y.X.); chenghua.sun@monash.edu (C.S.); 3 School of Materials Science and Engineering, Zhengzhou University, Zhengzhou 450001, China;

**Keywords:** composite film, barium titanate, resin, silane coupling agent, dielectric property

## Abstract

BaTiO_3_/polymer/Al (BPA) composite films for energy storage were fabricated by way of a roll coating and thermal curing process. The coating slurry consisted of silicon-containing heat-resistant resin (CYN-01) and BaTiO_3_ particles with various particle sizes obtained from commercial BaTiO_3_ powders processed at different durations of wet sand grinding in the presence of silane coupling agent (KH550), which not only improves the dielectric performance of the BPA films but also facilitates its production in a large scale. The major influence factors, such as the ratio between BaTiO_3_ and resin and the size of BaTiO_3_ particles, were investigated and their related mechanisms were discussed. The results show that modifying BaTiO_3_ particles (*D*_90_ = 0.83 μm) with the silane coupling agent of KH550 enhances the dielectric properties of the BPA films. The typical BPA films obtained exhibit a high dielectric constant of 32, a high break strength of 20.8 V/μm and a low dielectric loss of 0.014. The present work provides a simple and convenient way to prepare high-quality ceramic/polymer composite films for energy-storage application in a large scale.

## 1. Introduction

Capacitors with high energy and power densities are more and more indispensable in the present electronic information age [1]. Consumer electronic devices [2,3], smart and wearable electronics [4,5], aerospace industry [6], telecommunications equipment [7,8], the military industry [9] and the automobile industry [10,11], require high-performance capacitors and other energy-storage devices. To meet the demand for capacitors in the above applications, many capacitors with different types have been developed in recent years, including electrochemical capacitors (ECs) and dielectric capacitors. Electrochemical capacitors, including electric double layer capacitors (EDLCs) and pseudocapacitors (PCs), have attracted much attention owing to their high energy density, especially in terms of the electrochemical capacitors based on nanocarbon composites and 2D-materials [12,13]. The EDLCs based on carbon materials [14,15] and the PC based on metal oxides [16], metal oxides/graphene [17], MXenes [18] are achieved high energy density in recent reports. But the challenges in the power density and stability of ECs still lie ahead. Recently, the capacitors based on high energy-storage-density dielectric materials (e.g., ceramics) have attracted increasing attention because their ultrahigh power density, excellent charge/discharge capability and long lifetime [19]. For energy-storage applications, the dielectric materials are expected to possess high dielectric constants and breakdown strengths, low dielectric losses, good temperature stability and high flexibility for easy manufacturing [20]. However, it is still a great challenge for materials scientists to achieve the high dielectric materials with the above excellent overall properties. 

Composite dielectric films containing ferroelectric ceramic fillers and polymer matrixes have currently gained increasing attention, because these ceramics-based polymer composite films exhibit excellent dielectric properties and good flexibility as well as temperature stability [21]. For the ceramics-based polymer composite films, the properties of ceramic fillers, including their intrinsic dielectric properties, surface properties, size-distribution ranges, dimensions and morphologies, are the key factors that influence the performance of composite films and the resultant energy-storage devices [22]. Obviously, the intrinsic dielectric properties of the ceramic fillers have a direct impact on the dielectric performance of the composite films [23]. In addition, the size, morphology and surface characteristic of the fillers can affect the interfacial adhesion and dispersibility of the fillers in the polymer matrixes [24]. Therefore, the size-control and surface modification of ceramic fillers is vital to improve the dielectric performance of ceramics composite films.

The energy density (*U*_e_) of a dielectric material is defined as *U*_e_ = *∫E*d*D* and *D* = *εE*, where the *D, ε* and *E* are the electric displacement, dielectric permittivity and electric field, respectively [25]. In ceramics-based composite films, high-*ε* ceramic fillers, such as BaTiO_3_ (BT), SrTiO_3_, BaSrTiO_3_ and so on [26], are used to improve their dielectric permittivity and their breakdown strengths are usually modulated by adjusting the compatibility and monodispersity of the ceramic fillers in suitable polymer matrixes [27]. The aggregation of ceramic fillers and the defects existing at the interfaces between fillers and matrixes accelerate charges transmission, leading to a low breakdown strength. 

To enhance dielectric properties and energy-storage performance, there have been many reports on the control of the size and morphology of ceramic fillers, the compatibility and dispersibility of the inorganic fillers in the polymer matrixes. Chen et al. [28] modified the BT nanoparticles using a rigid liquid-crystalline fluoro-polymer to improve the compatibility between BT nanoparticles and the poly (vinylidene fluoride-trifluoroethylene-chlorotrifluoroethylene) matrix and found that the breakdown strength and energy density of the composite film obtained at the optimal conditions reached 514 kV/mm and 16.2 J/cm^3^, respectively. Xie et al. [29] reported a nanocomposite film consisting of poly-(dopamine)-modified BaSrTiO_3_ (PDA-modified BST) nanoparticles and poly(vinylidene fluoride) matrix and this film exhibited a dielectric constant of 35, a dielectric loss of 0.06 (at 100 Hz), a breakdown strength of 466 KV/mm and a high energy density of 11.0 J/cm^3^, indicating that the surface modification of BST with PDA remarkably enhances the compatibility of the two components and the structural homogeneity of the composite, which is the key to achieve high energy storage performance in composites films. Fu et al. [30] used BT nanorods (BTNRs) and BT nanoparticles (BTNPs) to fabricate BTNRs/PVDF and BTNPs/PVDF composite films and found that the BTNRs/PVDF film exhibits a higher dielectric permittivity than the BTNPs/PVDF due to the stronger internal electric field density distribution in the BTNRs/PVDF film. Zhu et al. [31] synthesized BT nanorods (BTNRs) by a microwave-assisted hydrothermal method and found that the BTNRs/epoxy composite film exhibits a high permittivity and good frequency stability. The above laboratory scale researches clearly indicate that the morphologies and surface modification of ceramic fillers highly influence the dielectric properties of the composite films and the energy-storage performance of the resultant capacitors. To achieve industrial scale applications, efficient and large-scale processes have to be developed to fabricate the ceramics/polymer films. Though the researches on the ceramics/polymer films for capacitor applications are multiple, the strategies suitable for industrial scale fabrication are still challengeable.

**Figure 1:**
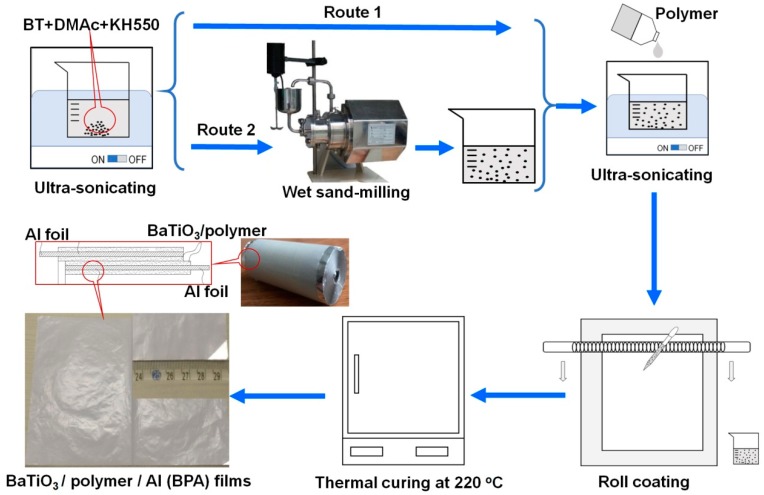
Schematic expression of the routes for the fabrication of BPA composite films.

In this work, we make a preliminary feasibility investigation on the roll-to-roll fabrication of BT/polymer composite films on the basis of the industrial coating machine using an Al foil as the flexible substrate. The possible factors that influence dielectric properties of the BT/polymer/Al (BPA) composite films, including the size-distribution of pristine BT powders, ultrafine-treating parameters and the ratios (*M*_BT_/*M*_Polymer_) of BT to polymer, are investigated systematically. The size and morphology control of the BT fillers is achieved by sand-milling and a silane coupling agent (i.e., KH550) is used to modify the BT surface and then enhance the compatibility between the BT fillers and polymer matrix. To imitate the industrial roll-to-roll coating process, we use a simple roll-coating method (see Figure 1) to fabricate BPA composite films, followed by a thermal curing. The typical BPA composite film obtained at the optimal conditions (*M*_BT_/*M*_Polymer_ = 4) exhibits a permittivity (*ε*), breakdown strength (*E*_b_) and loss (tan*δ*) of 32, 20.8 V/μm and 0.014, respectively.

## 2. Experimental

### 2.1. Chemicals and Setup

The polymer (ceramic glue), a silicon-containing heat-resistant resin, was purchased from the IPINRU Chen Yu Technology Co., Ltd (China, Product No. CYN-01 with a curing temperature of ~220 °C). BaTiO_3_ (BT, purity ≥ 99.9%) powders were supplied by Zhejiang Jiukang Electric Co., Ltd. (Wenzhou, China). A silane coupling agent (KH550, NH_2_CH_2_CH_2_CH_2_Si(OC_2_H_5_)_3_) was purchased from Guangzhou Yuantai Synthetic Material Co., Ltd and dimethylacetamide (DMAc) was purchased from Guangzhou Jinhuada Chemical Reagent Co., Ltd. Al foils (thickness = ~12 μm, tensile strength ≥ 180 MPa, ductility ≥ 15%) were purchased from Shenzhen Kejing Star Technology Co., Ltd. A sand miller (VB0.3Q, 0.3 mm of ZrO_2_ beads) was purchased from Suzhou Weige Nano Technology Co., Ltd. A bar coater (XT-300CA) and coating rod (D10-OSP010-L0400) were purchased from Shijiazhuang Ospchina Machinery Technology Co., Ltd. A high temperature blast drying oven (DHG-9079A) was purchased from Shanghai Heheng Instrument Equipment Co., Ltd.

### 2.2. Fabrication of BaTiO_3_/Polymer/Al Films

The routes for the fabrication of BPA composite films are presented in Figure 1. Typically, the pristine BT powders and DMAc were mixed with a mass ratio of BT/DMAc = 2.2 and then the silane coupling agent (KH550) with an amount of 8 wt.% of BT powders was added to the above mixture, followed by an ultrasonic dispersion for 30 minutes. There were two routes to fabricate BPA films: Route 1 without sand-milling and Route 2 with sand-milling treatment. 

In Route 1, after BT powders were uniformly dispersed in the DMAc/KH550 solution, a given amount of the binder polymer (CYN-01) was added to the DMAc/KH550/BT mixture and dispersed via ultrasonic treating for another 30 min to form a slurry containing BT and polymer. Then, a certain amount of the slurry was coated on an Al foil by a roll coater, followed by a curing treatment at 220 °C for 10 min and the BPA composite films were fabricated by Route 1. For Route 2, before the addition of the polymer (CYN-01), the DMAc/KH550/BT mixture was ultra-fined by the wet sand-milling for various times (1–60 min). The other processes for the fabrication of BPA composite films were similar to Route 1 (Figure 1).

### 2.3. Characterization and Dielectric Property Test

The X-ray diffraction (XRD) patterns of the BPA composite films and BT powders were recorded by a DX-2700BH X-ray diffractometer (Dandong Haoyuan, China) using Cu Kα irradiation. The morphologies of the BPA composite films and BT powders were observed using a TM3030Plus electron microscope (Hitachi, Tokyo, Japan). The dielectric constant (*ε*) and loss (tan*δ*) of the BPA films were measured using a high-precision high-voltage capacitor bridge (QS89, Shanghai Yanggao Capacitor Co., Ltd., Shanghai, China) and the dielectric performance test frequency was kept at 10 Hz. The breakdown strengths of the BPA films were measured using a withstand voltage tester (GY2670A, Guangzhou Zhizhibao Electronic Instrument Co., Ltd., Guangzhou, China). The particle sizes of the BT powders were measured using a laser particle size analyzer (LS-POP(VI), Omec Instruments Co., Ltd., Zhuhai, China). The film thickness of the BPA films was measured using a thousand fractions digital display of film sheet bench thickness gauge (CH-12.7-STSX, Shanghai Liuling Instrument Factory, Shanghai, China).

## 3. Results and Discussion

### 3.1. Fabrication of BPA Composite Films

**Figure 2:**
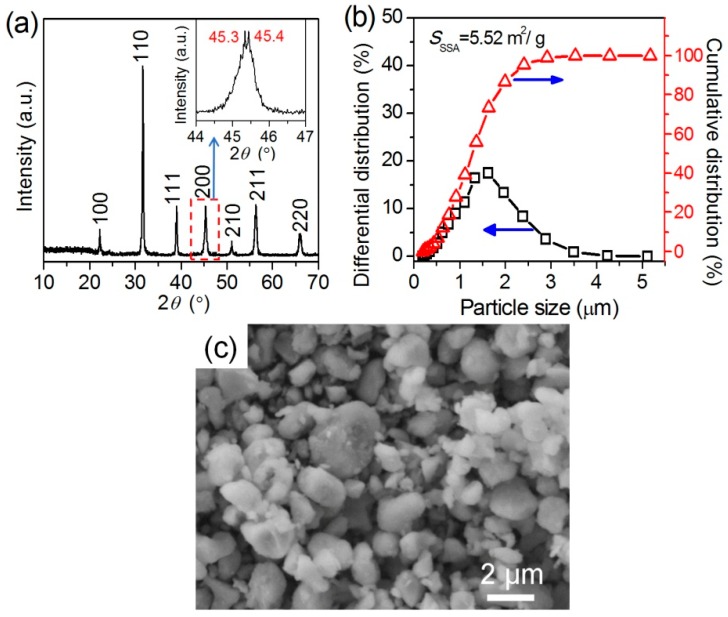
(**a**) X-ray diffraction (XRD) pattern, (**b**) particle-size distributions and (**c**) typical scanning electron microscopy (SEM) image of the pristine BT powders.

The BT powders used for the fabrication of BPA composite films were synthesized via a solid reaction using BaCO_3_ and TiO_2_ as the reactants at 1300 °C. Figure 2 shows the XRD pattern, particle-size distribution and the typical SEM image of the pristine BT powders. As the XRD pattern in Figure 2a shows, the distinct peaks at 2*θ* = 22.12°, 31.52°, 38.81°, 50.80°, 56.10° and 65.76° correspond to the (100), (110), (111), (210), (211) and (220) reflections of a cubic phase BT sample, respectively, according to the JCPDS card (no. 31–0174). The peak at around 45° consists of two sub-peaks at 45.3° and 45.4°, which can be attributed to (200) and (002) reflections of a tetragonal BT phase, respectively, according to the JCPDS card no. 05-0626 [32]. It may indicate that the BT sample may be a mixture of major cubic BT and minor tetragonal BT. Figure 2b shows the particle-size distribution plots of the pristine BT powders. The *D*_90_ of the pristine BT powders is about 2.08 μm and its specific surface area is 5.52 m^2^/g. From the differential distribution curve, one can see that the particle size of the BT powders is concentrated at around 1.6 μm. The SEM image of the pristine BT powders in Figure 2c shows that most of the BT particles are spherical but their sizes are not uniform. The sizes of most of the BT particles are less than 2 μm, which agrees with the particles-size result analyzed using the laser size analyzer.

In the DMAc/KH550/BT suspension, KH550 acts as both a dispersing agent and a surface hydrophobic treatment agent to improve the dispersibility and compatibility of BT powders in the DMAc solution and polymer matrix. The adhesion between BT/polymer composites and Al foils is excellent because of the coupling agent of KH550. The as-obtained BPA composite films after a curing treatment at 220 °C are gray, flexible and easy to be winded to form a cylindrical capacitor (see Figure 1). 

During the fabrication, the effects of surface modification (i.e., particle size) of BT powders by sand-milling treatment and mass ratios of BT to polymer on the dielectric properties of BPA composite films are systematically investigated via two routes with/without sand-milling treatment.

### 3.2. BaTiO_3_/Polymer/Al Composite Films with Pristine BaTiO_3_ Powders

We firstly investigated the suitability of the pristine BT powders in the fabrication of composite BPA films via Route 1 (Figure 1). The pristine BT powders are of a *D*_90_ of 2.08 μm with a specific surface area of 5.52 m^2^/g, as shown as Figure 2. The pristine BT powders are directly used to fabricate the BPA films with various *M*_BT_/*M*_Polymer_ values using a small amount of KH550 as the coupling and dispersing agent. 

**Figure 3:**
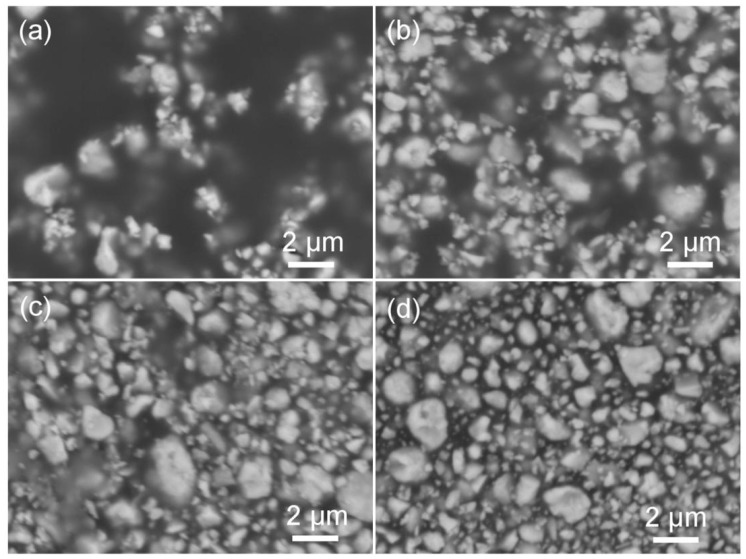
Typical SEM images of the BPA films fabricated via Route 1 with various BT/polymer mass ratios (*M*_BT_/*M*_polymer_): (**a**) 2, (**b**) 3, (**c**) 4 and (**d**) 5.

The distribution of BT particles in the BPA films were observed using SEM and Figure 3 shows the typical SEM images of the BPA films prepared via Route 1 with various mass ratios of BT/polymer (*M*_BT_/*M*_Polymer_). In the SEM images, the bright spots are BT particles and dark areas are polymer matrixes. As the value of *M*_BT_/*M*_Polymer_ increases from 2 to 5, one can find that the BT particles become denser and denser. In Figure 3a, there are many areas without BT particles due to the low *M*_BT_/*M*_Polymer_ value of 2. When *M*_BT_/*M*_Polymer_ = 3, there are some small areas without BT particles as shown as Figure 3b. As the *M*_BT_/*M*_Polymer_ ratio reaches 4, the as-obtained BPA film is uniformly distributed with BT particles in the polymer matrix (Figure 3c). Figure 3d shows a typical SEM image of the BPA film with *M*_BT_/*M*_Polymer_ = 5 and the BT particles are also uniformly distributed in the BPA film. When comparing the two BPA films with *M*_BT_/*M*_Polymer_ of 4 and 5, one can find that the particle density in the BPA films is similar but the edges of the BT particles in the BPA film with *M*_BT_/*M*_Polymer_ = 5 are clearer than those of the BPA film with *M*_BT_/*M*_Polymer_ = 4. This point may be explained by the suitable interaction between BT powders and ceramic glue and more BT particles with a smaller amount of polymer lead to incomplete encapsulation. Therefore, the optimum ratio of *M*_BT_/*M*_Polymer_ should be 4 for the formation of BPA films.

**Figure 4:**
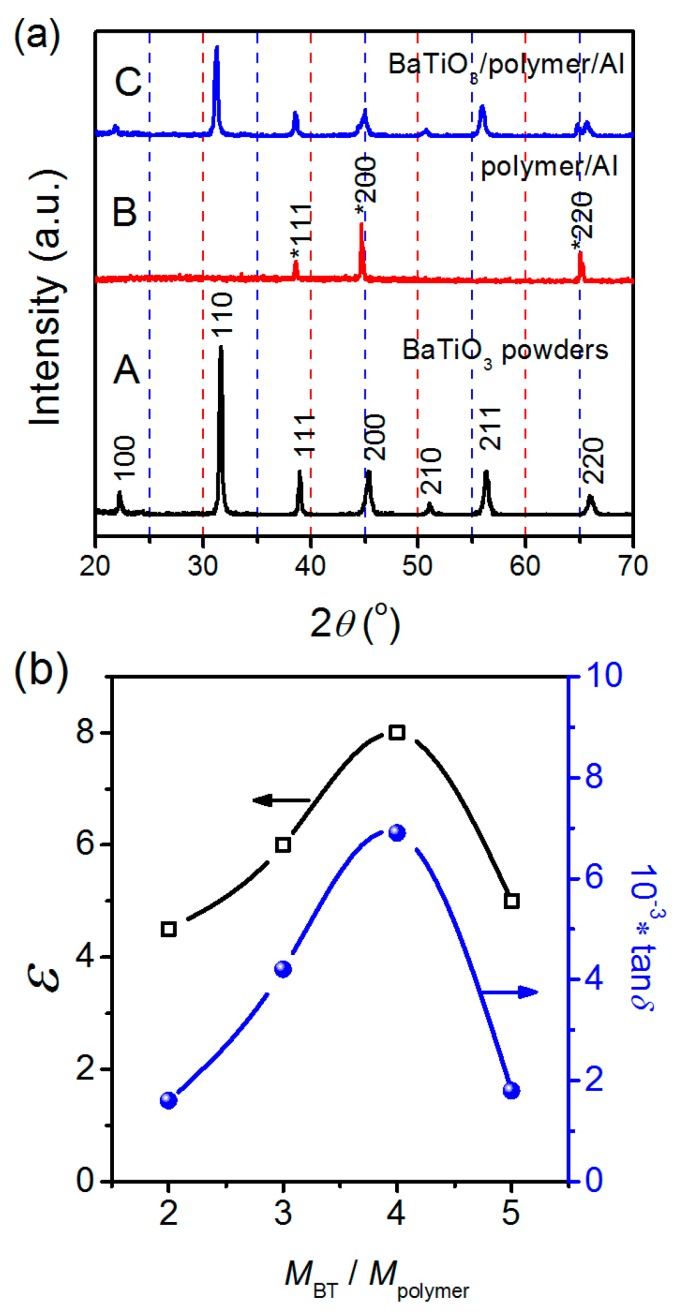
(**a**) Typical XRD patterns of the pristine BT powders, polymer/Al foil and BPA film (*M*_BT_/*M*_polymer_ = 4); (**b**) Plots of dielectric constant (*ε*) and dielectric loss (tan*δ*) of BPA films obtained via Route 1 as a function of BT/polymer mass ratios (*M*_BT_/*M*_polymer_).

Figure 4a shows the XRD patterns of the pristine BT powders, polymer/Al foil and BPA film (*M*_BT_/*M*_polymer_ = 4), determining the existence of the BT powders and Al foil. One can see that the BPA film (pattern C) is the superimposition of the pristine BT powders (pattern A) and Al foil (pattern B) and no other impurities can be found in the BPA film. The polymer (i.e., ceramic glue) is amorphous and no diffraction peaks belonging to polymer occur.

Figure 4b shows the dielectric constants and losses of the BPA films as a function of *M*_BT_/*M*_polymer_. One can see that as the *M*_BT_/*M*_polymer_ ratio increases from 2 to 4, the dielectric constant and loss show a monotone increase. When *M*_BT_/*M*_polymer_ = 4, the BPA film exhibits high dielectric constant of 8 and a dielectric loss (tan*δ*) of 0.0062. But when the *M*_BT_/*M*_polymer_ increases to 5, the dielectric constant and loss of the BPA film decrease sharply. This decrease may relate to the interaction and interface between BT powders and polymer species (refer to Figure 3). In general, in order to obtain a high dielectric constant, a common method is to increase the amount of ceramic fillers but at the same time the dielectric loss also increases obviously [33]. The possible reason should be that the higher filler amount in the BPA composite film, to some extent, reduces the bonding force between the BT powders and the electrode Al foil, as well as generates more pores and defects [34], leading to a larger dielectric loss. It should be noted that the dielectric constant of the BPA film is as low as 8 even at its optimum composition and we have to seek other means to improve the dielectric properties of the BPA films.

### 3.3. BaTiO_3_/Polymer/Al Composite Films with Ultra-Fine BaTiO_3_ Powders

**Figure 5:**
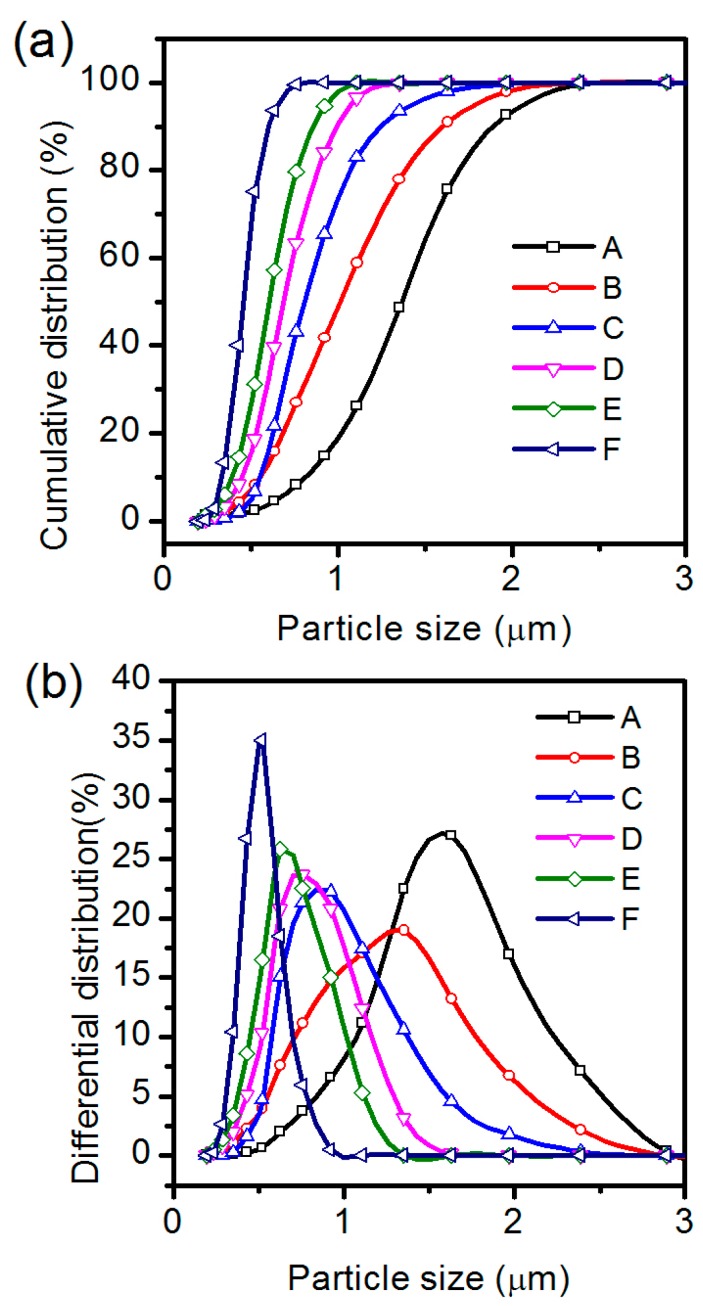
(**a**) Cumulative particle-size distribution curves and (**b**) differential particle-size distribution curves of the BT powders prepared by sand-milling the pristine BT sample for various times (t/min): (A) 1 min, (B) 10 min, (C) 20 min, (D) 30 min, (E) 40 min and (F) 60 min.

To improve the dielectric properties of the BPA composite films, the pristine BT powders were ultra-fined for various times using a wet sand-milling method, because the sand-milling is an efficient method to produce superfine powders in an industrial scale. Figure 5 shows the cumulative and differential particle-size distribution plots of the BT powders obtained after sand-milling the pristine BT powders for different times (*t* = 1~60 min). From the cumulative particle-size distribution plots (Figure 5a), we can determine the *D*_90_ values of the BT powders after sand-milling for various times. When the sand-milling time is 1, 10, 20, 30, 40 and 60 min, the *D*_90_ values of the BT powders obtained is 1.90, 1.60, 1.27, 0.99, 0.83 and 0.61 μm, respectively. Figure 5b shows the differential particle-size distribution plots of the BT powders obtained after sand-milling for various times. One can see that the particle-size distribution plots of the BT samples roughly accord with a normal distribution and that the smaller the *D*_90_ value is, the narrower the particle-size distribution range is. The regular particle-size change with the sand-milling time suggests that the wet sand-milling is an effective way to ultra-fine the pristine BT powders.

**Figure 6:**
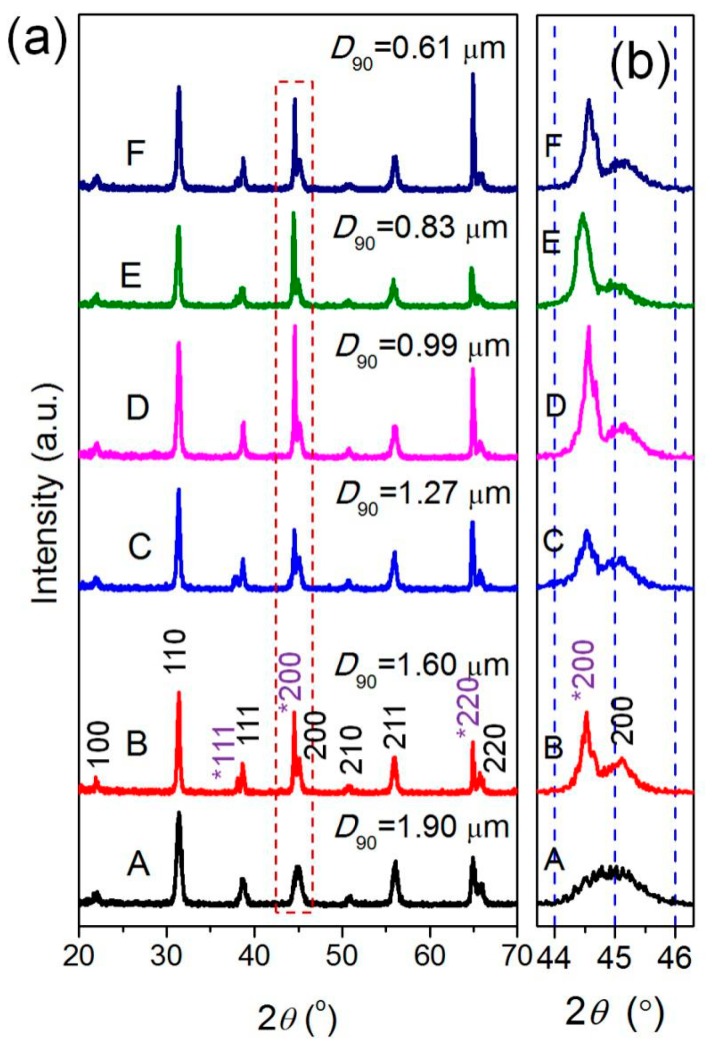
(**a**) XRD patterns of the BPA films obtained via Route 2 using BT powders with various particle sizes as the functional inorganic filler (*M*_BT_/*M*_polymer_ = 4); (**b**) the partial enlarged XRD patterns in the 2*θ* range of 45–46°.

The BT powders with various sand-milling times were used to fabricate BPA composite films via Route 2. Figure 6 shows the typical XRD patterns of the BPA films (*M*_BT_/*M*_polymer_ = 4) containing ultra-fine BT powders with various *D*_90_ values (0.61−1.90 μm). One can see that all the BPA films exhibit similar XRD peaks, belonging to the cubic BT phase and cubic Al phase (Figure 6a). The local enlarged XRD patterns at 2*θ* = 44–46° are shown in Figure 6b. One can see that the (200) diffraction peak of Al becomes weaker and weaker (or even disappear) with the increase in the particle size (*D*_90_) of the BT powders from 0.61 to 1.9 μm. This phenomenon may be resulted from the gradual increase in the thickness of the BPA films and the Al foil cannot be detected by XRD in the BPA films with large-particle BT powders. 

**Figure 7:**
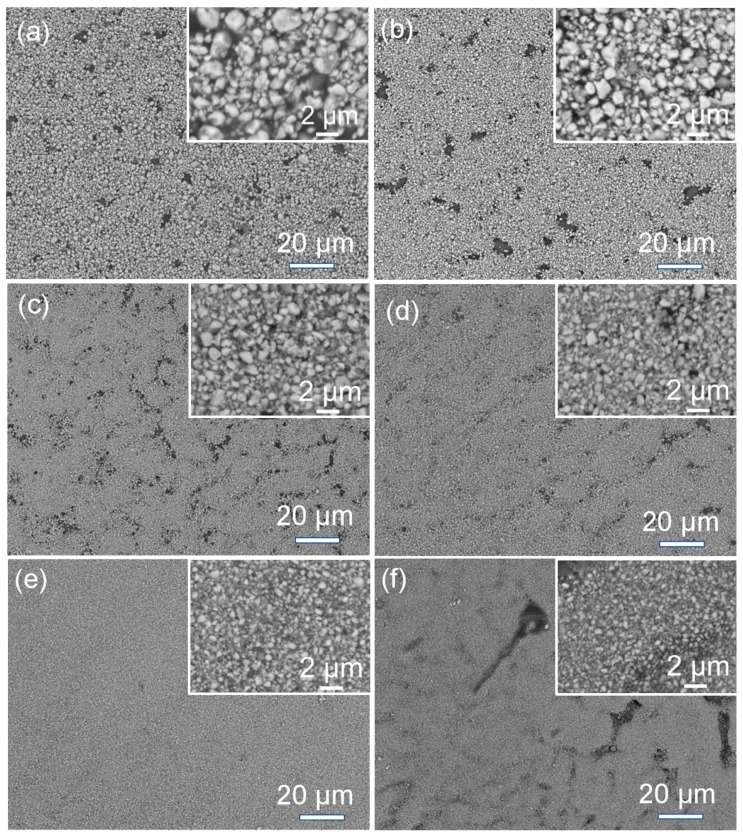
SEM images of the BPA films obtained via Route 2 using BT powders with various particle sizes as the functional inorganic filler (*M*_BT_/*M*_polymer_ = 4): (**a**) *D*_90_ = 1.9 μm, (**b**) *D*_90_ = 1.6 μm, (**c**) *D*_90_ = 1.27 μm, (**d**) *D*_90_ = 0.99 μm, (**e**) *D*_90_ = 0.83 μm and (**f**) *D*_90_ = 0.61 μm.

The morphologies and the distribution of the BT particles in the BPA composite films were further characterized using the SEM technique. Figure 7 shows the typical SEM images of the BPA films (*M*_BT_/*M*_polymer_ = 4) containing the BT powders with various particle sizes (*D*_90_ = 1.9, 1.6, 1.27, 0.99, 0.83 and 0.61 μm). The insets of the SEM images are the corresponding local high-magnification SEM images. There are many large holes with a diameter of 2 ~ 10 μm in the BPA composite films, when the particle size (*D*_90_) of the BT powders used is larger than 1.0 μm (e.g., 1.9, 1.6 and 1.27 μm for Figure 7a–c, respectively). The larger voids and openings occur with larger BT particles in the BPA films (Figure 7a–c). As Figure 7d,e shows, when the particle sizes (*D*_90_) of the BT powders are smaller than 1.0 μm, for example, 0.99 and 0.83 μm, the as-obtained BPA films become denser and more uniform. The holes gradually dwindle and even disappear and a dense and smooth BPA film (Figure 7e) is achieved when the *D*_90_ of the BT powder is 0.83 μm. However, when the size (*D*_90_) of the BT fillers decreases to 0.61 μm, some cracks occur in the BPA film, as shown as Figure 7f. These cracks in Figure 7f may be resulted from the aggregation of small BT powders in the BT/polymer composite. The other possible reason may be the unsuitable ratio of BT to polymer and smaller BT powders require a larger amount of polymer to form a uniform and fully infiltrated slurry. 

When comparing with the BPA film (*M*_BT_/*M*_polymer_ = 4) fabricated via Route 1 (Figure 3c), one can see that the BPA films fabricated via Route 2 exhibit smoother and denser morphologies than those of the BPA films fabricated via Route 1, especially the BPA film filled with BT nanoparticles with *D*_90_ = 0.83 μm (Figure 7e). The results indicate that the wet sand-milling is an effective way to reduce the sizes of the BT particles and narrow their particle-size distribution, which is important to achieve a smooth and dense BPA film. According to Figure 6 and Figure 7, the suitable BT size (e.g., 0.83 μm) is the key factor to form a dense and smooth BPA film.

**Figure 8:**
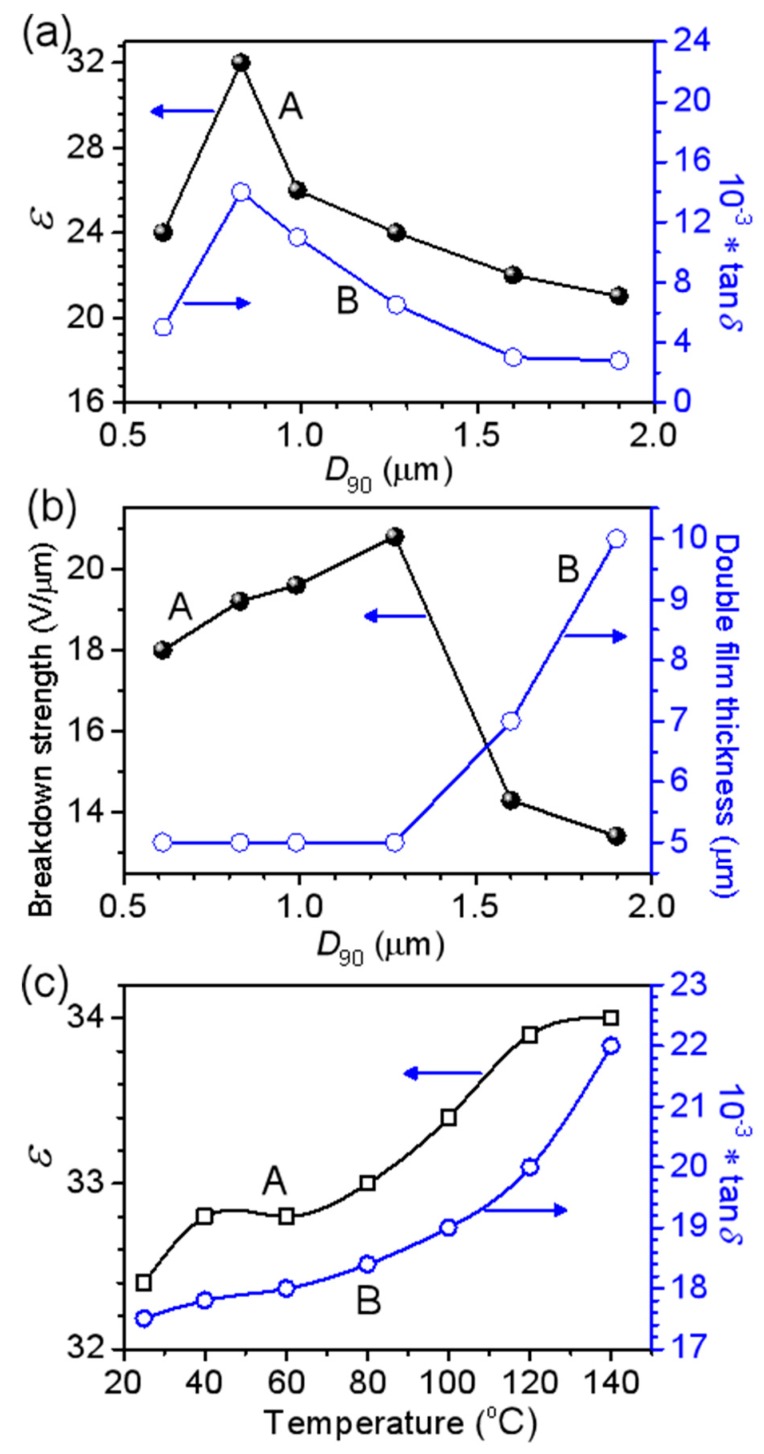
(**a**) Dielectric constant and loss of the BPA films obtained via Route 2 as a function of BT particle size (*M*_BT_/*M*_polymer_ = 4); (**b**) Breakdown strength and film double layer thickness of the BPA films as a function of BT particle size (*M*_BT_/*M*_polymer_ = 4); (**c**) Dielectric constant and loss of the BPA films obtained via Route 2 as a function of testing temperature (*M*_BT_/*M*_polymer_ = 4, *D*_90_ = 0.83 μm).

The dielectric properties of the BPA films containing BT powders with various particle sizes are shown in Figure 8. Figure 8a shows the change of the dielectric constant and loss as a function of the BT particle size (*D*_90_). One can see that the BPA film containing the BT powders with a *D*_90_ of 0.83 μm exhibits the highest permittivity of 32 and the other BPA films with larger or smaller *D*_90_ values show lower permittivity. The dielectric loss (tan*δ*) shows a change trend similar to their permittivity. For the BPA film with *D*_90_ = 0.83 μm, its tan*δ* is about 0.014. The change of the breakdown strength and film thickness versus *D*_90_ of BT powders is shown in Figure 8b. When the BT particle size increases from 0.61 to 1.27 μm, the thickness of the BPA film is kept at about 5 μm and its breakdown strength is slightly enhanced from 18 to 21 V/μm. When the size of BT particles increases from 1.27 to 1.9 μm, the as-obtained BPA film becomes thicker and thicker, increasing rapidly from 5 to 10 μm in thickness; whereas, its breakdown strength sharply drops from 21 to 5 V/μm. As Figure 8a,b shows, the BPA composite film with BT of *D*_90_ = 0.83 μm exhibits the largest permittivity of 32 and the BPA composite film with BT of *D*_90_ = 1.27 μm has the highest breakdown strength of 21 V/μm. When comparing Figure 8 with Figure 4, we find that the dielectric properties of the BPA films fabricated via Route 2 are obviously higher than those of the BPA films fabricated via Route 1, indicating the wet sand-milling is a crucial strategy to improve the dielectric performance of the BPA films due to the more uniform and dense distribution of BT powders. 

Table 1 compares the dielectric properties of some ceramic/polymer composite films published recently. One can find that the BPA film prepared by the simple roll-coating method in this work shows good comprehensive dielectric properties (i.e., high dielectric constant and low dielectric loss). The high dielectric performance of the BPA films should be attributed to the dense and uniform arrangement of the BT particles, resulting from the fine BT particles with a narrow size distribution and the high compatibility between BT particles and polymer matrix. The sizes of BT particles prepared by wet sand grinding are more uniform than those obtained via other methods in previous works reported. On the other hand, the BT particles in the special resin CYN-01, produced by IPINRU Chen Yu Technology Co., Ltd. (Shenzhen, China), exhibits more compatible than those in PVDF. So, the dielectric performance of BPA film in this work is better than the others in previous works.

Figure 8c shows the change in the dielectric constant and loss of the BPA film as a function of testing temperature (*M*_BT_/*M*_polymer_ = 4, *D*_90_ = 0.83 μm). One can see that the BPA film shows stable dielectric properties in different test temperature and the dielectric constant and loss exhibit little fluctuation when the test temperature increases from 20 °C to 140 °C.

Taking Figure 7 and Figure 8 into account, the particle size and distribution of BT powders have a tight effect on the dielectric properties of the corresponding BPA films obtained via Route 2. The BT powders with a *D*_90_ of 0.83 μm obtained by wet sand-grinding for 40 min exhibit the highest dielectric properties in the present test conditions. These results are helpful in the fabrication of high-quality energy-storage devices on the bases of BT ceramic powders.

## 4. Conclusions

In summary, a simple roll-coating and thermal curing process has been developed to fabricate BT/polymer composite films on Al foils for energy-storage capacitor applications. The particle sizes of the BT powders are conveniently modulated by the wet sand-milling treatment and the particle sizes of the BT powders have obvious effects on the dielectric properties of the as-obtained BPA composite films. The dielectric constant, break strength and dielectric loss of the BPA films can be efficiently regulated by the BT fillers with various particle sizes (*D*_90_). Under the optimal conditions, the BPAfilm containing BT with *D*_90_= 0.83 μm exhibits a high dielectric constant of 32, a high break strength of 20.8 V/μm and a low dielectric loss of 0.014, being close to or beyond the results reported recently. This work provides a simple but industrial strategy to fabricate ceramic/polymer composite films for energy-storage capacitor applications.

## Figures and Tables

**Table 1:** Comparisons of dielectric constant, dielectric loss and breakdown strength of the composites containing BT particles.

Fillers	Polymer Matrix	Dielectric Constant	Dielectric Loss	Breakdown Strength	Ref.
BT microparticles	Resin	32	0.014	20.8 V/μm	This work
BT nanoparticles (60 nm)	In suit prepared polyimide	3.85	0.0024	334.26 kV/mm	[35]
PDA coated BT nanoparticles (100 nm)/BN nanosheets	Poly(vinylidene fluoride-chlorotrifluoroe thylene)	11.7	0.10	425 MV/m	[36]
PVP coated BT nanoparticles (100 nm)	Poly(vinylidene fluoride)	80.4	0.085	240 kV/mm	[37]
BT@Al_2_O_3_ nanoparticles (150 nm)	Poly(vinylidene fluoride)	17.5	0.02	312 kV/mm	[38]
Sphere-like TiO_2_ nanowire clusters	Poly(vinylidene fluoride-co-hexafluoropylene)	11.9	0.048	160 kV/mm	[39]
Dopamine modified urchin-like hierarchical structure of BT particles @ TiO_2_ nanowires	Poly(vinylidene fluoride-co-hexafluoropylene)	14.7	0.0037	240 kV/mm	[40]
Dopamine-coated BT@SiO_2_ nanofiber	In suit prepared polyimide	5.05	0.0225	346 kV/mm	[41]
Ethylene propylene diene monomer coated BT nanoparticles (<100 nm)	Polypropylene	5.8	/	370 MV/m	[42]
CaCu_3_Ti_4_O_12_@TiO_2_ nanofibers	In suit prepared polyimide	5.85	0.025	236 kV/mm	[43]
BT modified with 2-phosphonobutane-1,2,4-tricarboxylic acid	In suit prepared polyimide	23.5	0.00942	80 MV/m	[44]
BT/TH-615 acrylic-acrylate-amide copolymer	In suit prepared polyimide	20.3	0.00571	73 MV/m	[44]
